# Suspension polymerization of bioelectronic interfaces on living cells

**DOI:** 10.1039/d5mh02264a

**Published:** 2026-03-26

**Authors:** Hanne Biesmans, Charlotte Theunis, Rebecka Rilemark, Caroline Lindholm, Marle E.J. Vleugels, Tobias Abrahamsson, Xenofon Strakosas, Jennifer Y. Gerasimov, Daniel T. Simon, Magnus Berggren, Eva Olsson, Chiara Musumeci

**Affiliations:** a Laboratory of Organic Electronics, Department of Science and Technology, Linköping University 601 74 Norrköping Sweden daniel.simon@liu.se chiara.musumeci@liu.se; b Department of Physics, Chalmers University of Technology 41296 Göteborg Sweden; c Wallenberg Initiative Materials Science for Sustainability, Department of Science and Technology, Linköping University 601 74, Norrköping Sweden

## Abstract

The development of robust and biocompatible interfaces between living cells and electronic devices is essential for the advancement of bioelectronic and medical technologies. Organic conjugated polymers have emerged as promising materials for this purpose owing to their mixed ion and electron conductivity, as well as their mechanical and chemical flexibility. Here, we present a simple, genetic-modification-free protocol for enzyme-mediated, cell-templated polymerization that enables the formation of conductive polymer coatings on the surface of living cells. By exploiting the non-specific adsorption of horseradish peroxidase (HRP) onto the cell membrane followed by *in situ* suspension polymerization of a thiophene-based monomer, we achieve localized polymeric coatings on the cell membrane without compromising cell viability or excitability. The method is successfully applied to different cell lines, and the polymer properties are successfully characterized by absorption spectroscopy, scanning electron microscopy, and conductive atomic force microscopy. Functional assays demonstrate preserved cellular responsiveness and viability, and the polymer coating remains stable for up to four days. This *in situ* polymerization approach offers a rapid, versatile, and minimally invasive strategy for engineering bioelectronic interfaces, expanding the toolkit for integrating electronics with living systems.

## Abstract 2

This work introduces an easy method to coat living cells with conductive polymers using enzyme-mediated polymerization. The resulting coatings are conductive and cytocompatible, offering a minimally invasive way to build bioelectronic interfaces.

## Graphical abstract

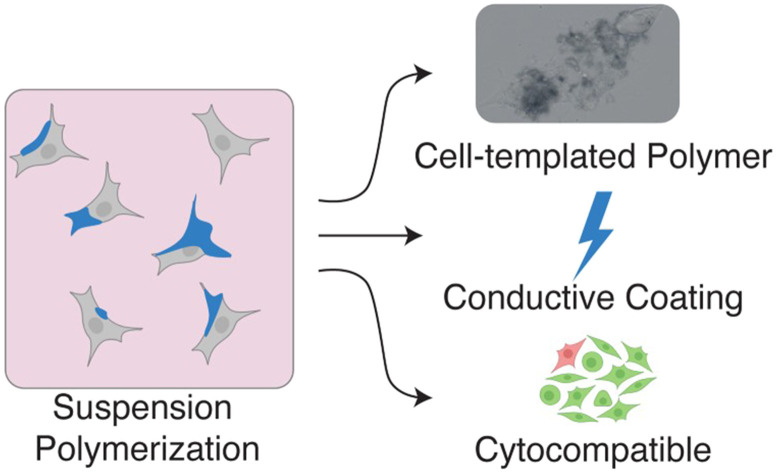

**New concepts**

Creating electrical interfaces directly at the level of individual cells has the potential to be highly transformative for bioelectronics, yet no broadly accessible method currently exists to achieve this. Most approaches rely on rigid device structures, patterned substrates, multistep protocols or genetic engineering to localize conductive materials at the cell surface. Here, we present a simple suspension-based strategy in which a mild enzymatic reaction generates a conductive polymer layer directly on living cells. By allowing the cell membrane to define the geometry of the coating, this method achieves polymer growth without genetic modification or microfabrication. This concept offers a practical route to cell-specific electronic functionality and expand the toolkit for creating next-generation of soft, adaptive bioelectronic interfaces.

## Introduction

The seamless integration of biological systems with electronic devices represents a key challenge in the advancement of bioelectronic interfaces. As the demand grows for high-resolution neural recording, precise stimulation, and real-time biosensing, there is a pressing need for materials that can bridge the gap between the soft, dynamic environment of living cells and the rigid, often incompatible nature of engineered electronics.^1^

Organic conjugated polymers have emerged as promising candidates for these applications due to their mechanical and chemical flexibility, biocompatibility, and mixed ion electron conduction.^2^ Traditional approaches to integrating these polymers with biological systems have focused on coating pre-formed, (semi)rigid electrodes or embedding cells within polymer matrices. Chemical modifications of the surface or substrate patterning are common methods to improve cellular adherence to these traditional substrates. However, such configurations often limit spatial resolution and adaptability, and cannot achieve the precise, cell-level control required for selective stimulation or monitoring.^3,4^ The next step in organic bioelectronics lies in achieving direct, dynamic, and localized interfaces, where conjugated polymers are formed or assembled directly at the surface of individual living cells. Such approaches enable intimate electrical coupling without the mechanical mismatch or invasiveness of conventional electrode-based systems, allowing each cell to effectively serve as its own bioelectronic node.

A promising route toward this vision is *in situ* cell-templated polymerization, which leverages the cell membrane itself as a substrate to initiate polymer growth directly at the cell surface. This method enables cell-templated coatings, potentially overcoming the limitations of pre-fabricated electrode interfaces. The field of *in situ* polymerization for biological applications was initiated in 2007 by Martin's group, who demonstrated electropolymerization of poly(3,4-ethylenedioxythiophene) (PEDOT) around living cells to create cell-templated microelectrode coatings.^5–7^ Building on this foundational work, several research groups, including our own, have since explored enzyme-mediated polymerization to improve biocompatibility and achieve better interfaces in various organisms, including plants,^8,9^*Hydra vulgaris*,^10,11^ zebrafish,^12^ leeches,^13^ and mice.^14,15^ Further advances were made by Bao's and Deisseroth's teams, who genetically modified cells and *C. elegans* to express specific enzymes, achieving localized polymer formation and precise targeting.^16,17^ Subsequent introduction of light-induced polymerization strategies, using photosensitizer proteins, enabled polymer formation exclusively in illuminated regions.^18–20^

Our group recently developed a straightforward, genetic-modification-free protocol for anchoring conductive polymers onto synthetic and biological membranes.^21^ In this approach, an amphiphilic thiophene-based monomer functionalized with an oleyl moiety coupled with a poly(ethylene glycol) chain (ETE-PEGO) was first inserted into the cell membrane, followed by enzymatic copolymerization of a water-soluble analogue (sodium 4-(2-(2,5-bis(2,3-dihydrothieno[3,4-b][1,4]dioxin-5-yl)-3-thienyl)ethoxy)-1-butanesulfonic acid salt, from now on referred to as ETE-S) using horseradish peroxidase (HRP) and hydrogen peroxide (H_2_O_2_). The method, validated in both synthetic vesicles and living cells, produced conformal, conductive coatings without affecting cell viability or excitability. However, improvements in polymer stability and uniformity of cell coverage remain desirable.

**Fig. 1:**
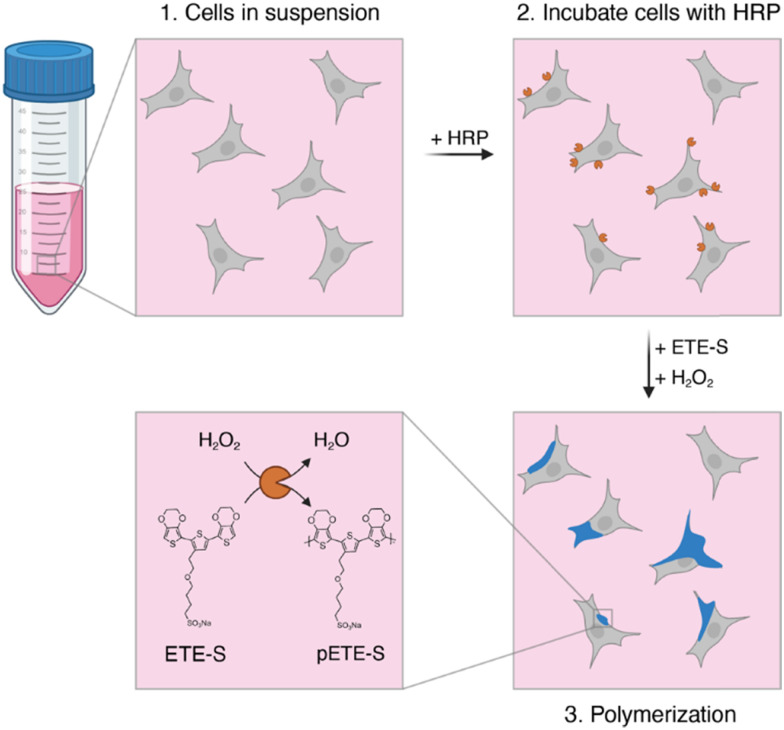
Schematic representation of *in situ* suspension polymerization. Cells in suspension are first incubated with HRP while shaking. Then unbound HRP is rinsed off and H_2_O_2_ and ETE-S are added to initiate localized polymerization. Figure made with BioRender.

In the present study, we introduce a simplified approach to cell-templated polymerization, without the need for genetic modification or an anchor molecule (see Table S1 for a detailed comparison). This method is not only rapid and straightforward, but is also performed in a suspension, which facilitates more consistently-formed conducting coatings across the entire cell surface. This two-step procedure involves initial incubation of cells with HRP under mild agitation to achieve non-specific adsorption of HRP to the cell membrane (Fig. 1). Subsequently, the addition of ETE-S monomers and H_2_O_2_ initiates the enzymatic polymerization process, resulting in the formation of conductive polymer localized at the cell membrane.

## Results and discussion

### Polymerization of pETE-S around the cell membrane

**Fig. 2:**
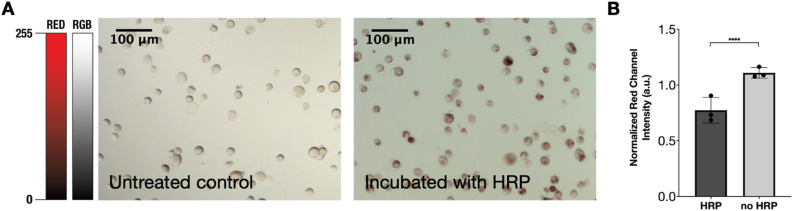
DAB staining. (A) F11 cells were incubated with HRP and subsequently exposed to DAB to stain cells with adsorbed HRP. Brown-tinted cells have HRP, which amounts to 51.3 ± 4.0% of the cells, whereas the control sample that was not exposed to HRP did not show any bound enzyme. Scale bars are 100 µm. (B) Quantitative analysis of red channel intensity in brightfield images. Areas with lower light intensity indicate HRP binding, as DAB deposition darkens the affected cells. Data sets were normalized *versus* untreated control samples. All data are presented as mean ± SD (*n* = 3 biological replicates, all averages of three technical replicates). All data were analyzed using a two-tailed *t*-test and **** = <0.0001.

First, HRP was immobilized on the cell membrane by incubating suspended cells with HRP (107.86 U mL^−1^) for 30 minutes under gentle agitation. HRP binding was confirmed using 3,3′-diaminobenzidine (DAB) staining. In this reaction, HRP catalyzes the oxidation of DAB, forming a semi-soluble reddish brown precipitate whose optical intensity is correlated to the amount of HRP present (Fig. 2A). Since DAB deposition reduces transparency, higher HRP levels correspond to lower color saturation. Accordingly, HRP-treated cells exhibited a significantly reduced red channel intensity, indicating increased HRP binding (Fig. 2B). Quantitative analysis further showed that approximately 51.3 ± 4.0% of the cells were confirmed to be coated with HRP (Fig. 2A).

**Fig. 3:**
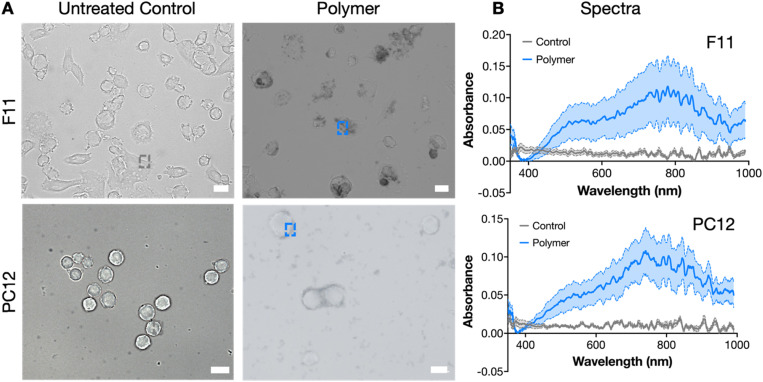
Visualization and characterization of cell-templated pETE-S. The polymerization protocol was tested on both F11 (top row) and PC12 (bottom row) cells. (A) Bright-field images where the left column shows untreated cells and the right column shows cells undergoing polymerization. Scale bars are 20 µm. (B) Spectroscopic analysis of samples in (A) showing the typical spectral signature of pETE-S confirming the nature of the dark structures in the right column of (A). The control spectra refer to untreated cells, and all data are presented as mean ± SD (average of 10 measurements).

Following HRP immobilization, unbound HRP was removed by centrifugation and the level of HRP adsorption was indirectly quantified by a 3,3′,5,5′-tetramethylbenzidine (TMB) assay on the supernatant (containing unbound HRP). We estimate that on average, 15% ± 4 of the initially added HRP was adsorbed to the cells, corresponding to 16.179 units per mL HRP. After removal of unbound HRP, enzymatic polymerization was initiated by adding a mixture of H_2_O_2_ and ETE-S. Subsequently, dark patches were visible in bright-field images all around and on cells (Fig. 3). This protocol was successfully reproduced and optimized in two cell lines: F11, a hybrid of rat embryonic dorsal root ganglion and mouse neuroblastoma cells, and PC12, an inducible neuron-like cell line from a rat adrenal pheochromocytoma. Additionally, it was reproduced (but not optimized) in Red Blood Cells (RBCs; Fig. S1) and SH-SY5Y cells (Fig. S2).

To further assess the selective polymerization at the surface of HRP-adsorbed cells, a control experiment was performed in which HRP, H_2_O_2_, and ETE-S were added simultaneously in a one-step reaction (Fig. S3). Three different HRP concentrations were tested: 107.86 units per mL (matching the initial HRP concentration of the original protocol), 10.79 units per mL and 21.58 units per mL (mimicking the concentrations known to participate in polymerization based on the TMB assay). These experiments showed that only minimal polymer formation occurred without the initial HRP incubation step, and polymerization was observed on the smallest-sized cells.

To evaluate the stability of the HRP interaction with the cell membrane, two additional control experiments were conducted. In the first, a two-hour delay was introduced between HRP incubation and polymerization to allow for establishing a new equilibrium between HRP adsorbed to the cell membrane and HRP dispersed in the fresh medium. In the second, an additional wash step was included after HRP incubation but before polymerization (Fig. S4). The delayed incubation and wash step both resulted in reduced cell-templated polymer formation, and increased amount of polymer patches not bound to cells. This suggests that the HRP binding to the cell membrane can be suppressed or disrupted by washing or extended incubation. This also suggests that the actual proportion of cells with adsorbed HRP is likely higher than the 51.3% estimated from DAB staining, since the staining protocol involved an extra wash-step after HRP exposure.

While the exact mechanism of membrane binding remains unclear, earlier studies from the late 1980s proposed that HRP may interact with glycosyltransferases on the cell membrane, including sialyltransferases, in various cell lines such as HeLa, rat pituitary cancer cells, and fibroblasts.^22–24^ Those studies also demonstrated that HRP binding is calcium-dependent.^24^ Based on this, increasing the calcium concentration in our medium could enhance binding efficiency. Furthermore, previous work also reported that HRP dissociates from the membrane after approximately 5 hours in calcium-free medium, which is consistent with our observations: when the delay between HRP incubation and polymerization exceeded 2 h in a medium with a low calcium concentration (0.30 mM), we observed a reduced number of cells coated with polymer and an increased formation of polymer aggregates not associated with the cell surface.^24^

### Characterization of the formed polymer

To characterize the nature of the dark structures formed around the cells, localized absorbance spectroscopy was performed, revealing the typical optical features of pETE-S in both its undoped (*λ* = 500–600 nm) and doped (*λ* > 600) forms^25^ (Fig. 3B). Significant variability in polymer deposition was observed across replicates, even under identical experimental conditions, suggesting that the coating process is influenced by subtle experimental variations, likely reflecting the inherent heterogeneity of cell cultures *in vitro* (Fig. S5).

**Fig. 4:**
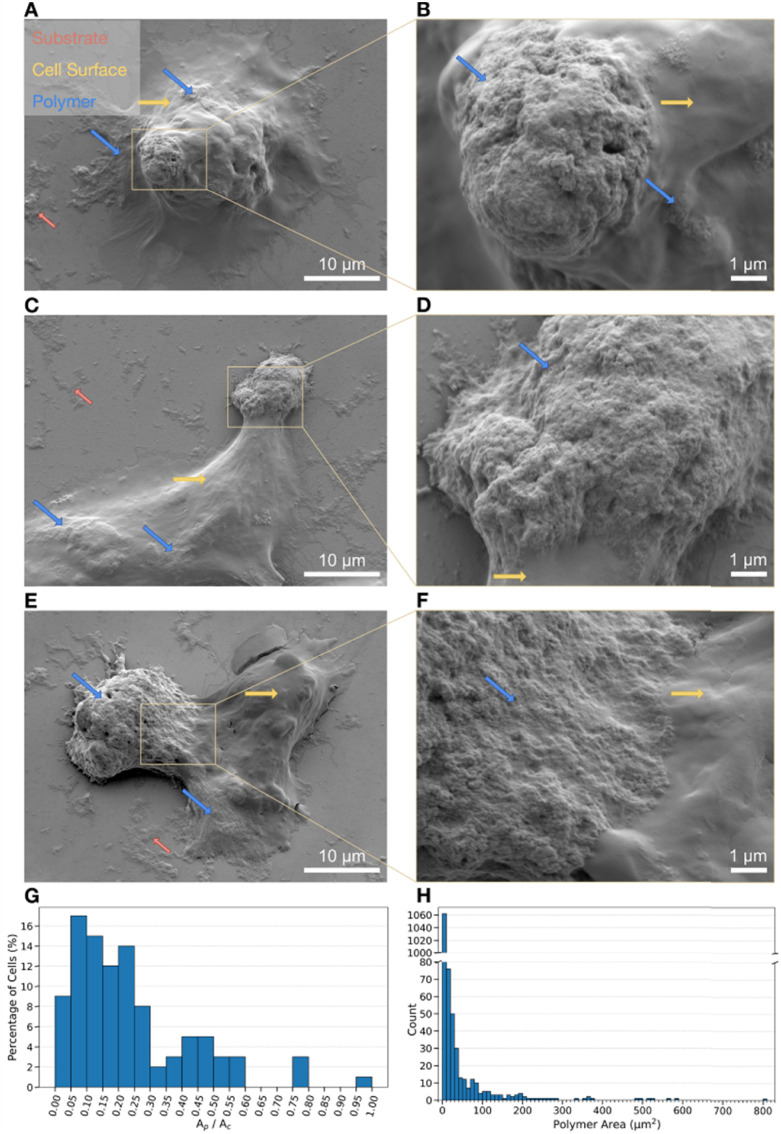
Surface morphology of F11 cells with pETE-S coatings. (A)–(F) SEM secondary electron images of cells with polymer layers that coat the cells in patches. The polymer patches (blue arrows) have a rougher surface morphology than the cell surface (yellow arrows). Polymer patches are also found on the surrounding substrate (red arrows). (G) Histogram of the ratio of the total projected area of all polymer patches in contact with the cell, Ap, and the projected cell area, Ac, obtained from SEM images of 100 cells. The average ratio of Ap/Ac is 0.24. (H) Size distribution of polymer patches. Most polymer patches are 0.01–10 µm^2^ but polymer patches up to 805 µm^2^ have been observed.

Scanning electron microscopy (SEM) secondary electron imaging of F11 cells after suspension polymerization revealed a polymer coating with a rough surface morphology, as shown in the regions marked with blue arrows in Fig. 4. The polymer coating was non-uniform and coated the cells in patches, which was also observed in the bright-field images in Fig. 3. This may be a result of uneven HRP adsorption as the DAB staining (Fig. 2) also appeared patchy in most cells. The bare cell surface without polymer (yellow arrows in Fig. 4) was significantly smoother in comparison. The chemical compositions of the two distinct surface morphologies attributed to the polymer patches and the bare cell surfaces were determined with energy dispersive X-ray spectroscopy (EDX) in the SEM (Figure S6). This analysis showed that the polymer regions contained more sulfur compared to the bare cell regions, which is attributed to the sulfonate group and thiophene backbone of pETE-S (Fig. S6A–D).

Polymer patches were observed on top of the cells, sometimes extending onto the underlying substrate, as well as patches scattered on the substrate without contact with the cells (Fig. 4 and Fig. S7). A similar patchy polymer coating with rough surface morphology was also observed for PC12 cells (Fig. S8). To quantify the size of the polymer regions contacting the cells, the cellular area and the area of all individual polymer patches contacting each cell were determined based on SEM images of 100 different F11 cells. This resulted in a total of 1330 measured polymer patches. Most polymer patches were in the range of 0.01 µm^2^ to 10 µm^2^, but polymer patches up to 805 µm^2^ were observed (Fig. 4H). The mean projected area of the cells was found to be approximately 970 µm^2^, corresponding to an average diameter of 35 µm if the cells were approximated to be round and flat. The ratio Ap/Ac between the total area of polymer patches, Ap, and the cellular area, Ac, was then below 0.60 for 96% of the cells (Fig. 4G). This indicates that the polymer coating rarely covered more than 60% of the cell surface. On average, the ratio Ap/Ac was 0.24, meaning that the polymer coverage was slightly less than 24%, since the ratio Ap/Ac also considers polymer areas extending outside the main cell body. It should be noted that the areas measured in the SEM images are projected areas. The true areas can be larger, especially for a cell with pronounced surface topography. Moreover, the statistical analysis considers only the top surface of the samples, as the SEM images do not provide information on the presence of polymer beneath the cells. When combined with the extensive washing of the samples during preparation, it is therefore highly likely that the reported 24% coverage represents an underestimation. Nevertheless, as shown in Table S1, this value is, to the best of our knowledge, among the highest reported levels of continuous polymer coverage achieved using comparable methods. In contrast, other approaches have been reported to produce polymer aggregates rather than patch-like coatings. In addition, partial coating avoids complete cellular encapsulation and allows sufficient diffusion of nutrients into the cell, thereby reducing potential cytotoxic effects of the polymer. While the reduced contact area may complicate electrical coupling with the cell, we consider this a reasonable trade-off that ultimately favours cell health and viability.

**Fig. 5:**
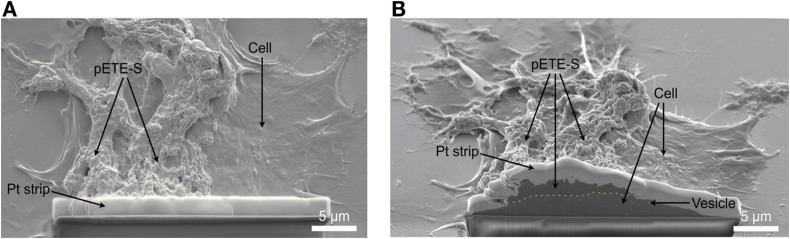
SEM secondary electron images visualizing the polymer patch geometry. The thickness of the polymer patch is revealed in a cross section prepared using FIB milling. A platinum strip is deposited to protect the surface morphology. (A) top view and (B) sample tilted 50° for imaging of the cross section surface. The polymer patch is located on top of the F11 cell surface (yellow dashed line in (B)). The thickness of the polymer patch in the cross section is in the range 0.5–2.3 µm. Vesicles can be seen inside the cell region as bright, round particles.

The thickness of the polymer coating varied between different patches and even within different parts of the same patch. To get an estimate of the thickness, cross sections of F11 cells with polymer coatings were prepared by focused ion beam (FIB) milling (Fig. 5). SEM imaging of the cross sections using backscattered electrons (Fig. S9) revealed multiple different contrast regions. The darkest region found near the top of the cross sections belonged to the polymer coating. EDX showed that this region also contained a higher amount of sulfur than the underlying cell (Fig. S9B). The thickness of this darker layer ranged from approximately 0.1 µm to 2.5 µm. Note that vesicles were observed in the surface morphology in SEM images of polymer-coated F11 cells (black arrows in Fig. S7) and untreated control cells (Fig. S10). This indicates that the vesicles form in the cells independently of the polymerization process.

**Fig. 6:**
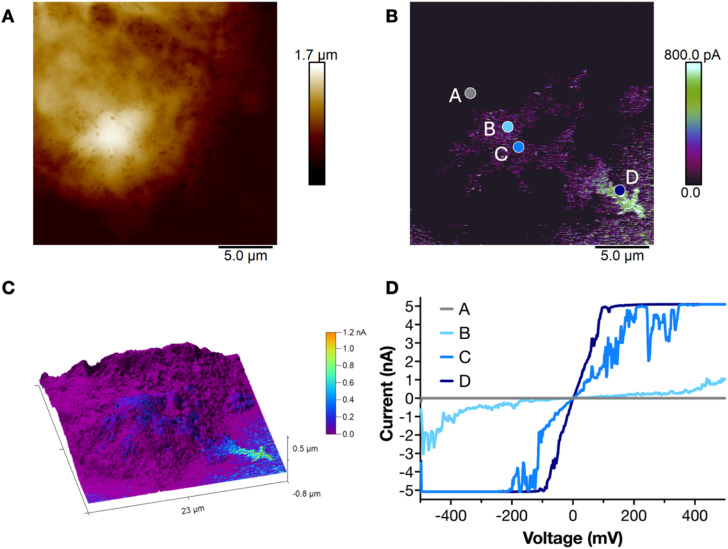
Electrical characterization of polymer on F11 cells through c-AFM. Topography (A) and current map (B) of a cell with assembled polymer. (C) 3D overlay of the topography image and the current map, showcasing the presence of a conductive region on top of the cell. (D) Local *I*–*V* curves measured on different corresponding points in (B).

To investigate the electrical properties of the formed polymers, we employed conductive atomic force microscopy (c-AFM). Following the polymerization process, treated F11 cells were seeded onto gold-coated coverslips and subsequently fixated and dried under a nitrogen stream. A metal-coated nanoscale probe was used to simultaneously acquire topographical and electrical characteristics at the single-cell level. The topography image (Fig. 6A) revealed a cell approximately 20 µm in diameter and 1 µm in height; however, it did not differentiate between cellular and polymeric regions. In contrast, the corresponding current map (Fig. 6B) highlighted a substantial conductive area coinciding with the cell region. Overlaying the topographical and current data (Fig. 6C) confirmed the presence of a conductive polymer both on the cell surface and extending onto the underlying substrate, as seen before in SEM. Localized current–voltage (*I*–*V*) measurements were performed at points A–D in Fig. 6B: point A corresponds to uncoated cell membrane, points B and C to polymer-coated regions on the cell and point D to a polymer region in direct contact with the gold substrate. Measured currents on the order of several nA further confirmed the conductive nature of the *in situ* formed polymer. The magnitude of the measured current increased the closer we measured to the gold substrate due to better contact. Due to the inherent difficulty of forming reliable macroscopic electrical contacts to soft electrodes, electrical characterization in this study was restricted to c-AFM measurements.

### Characterization of cells after polymerization

**Fig. 7:**
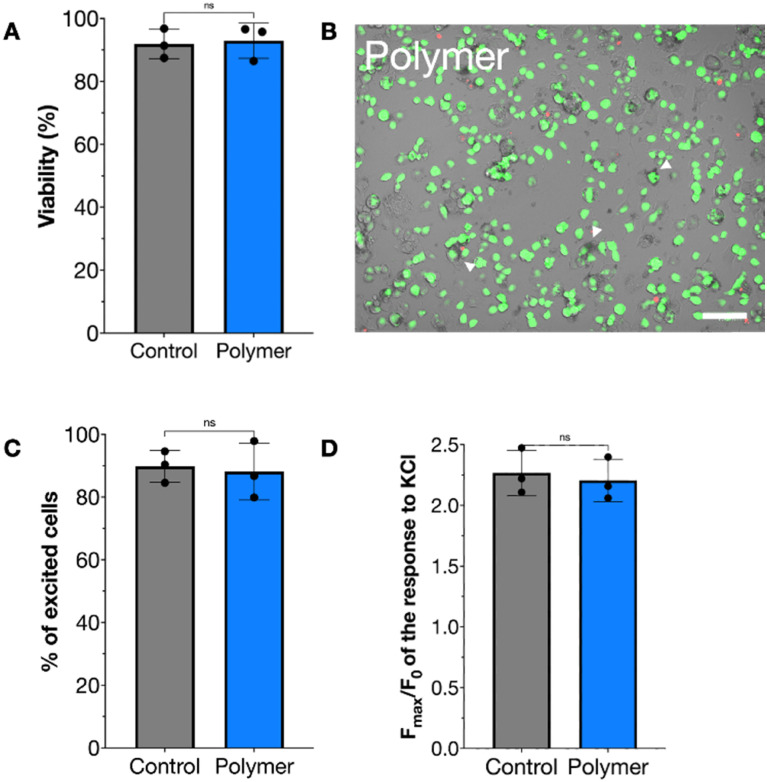
Viability and calcium imaging of cells after polymerization, determined using a fluorescence viability kit in which calcein (green) stains the living cells, while ethidium homodimer–1 (red) intercalates in the DNA of dead cells. (A) Viability of F11 cells. (B) Representative image showing polymer annotated with white arrows. Scale bar 100 µm. Calcium imaging showed the percentage of cells that were excited by KCl (C) and the calcium responses both in the control group and in the polymer group (D). All data are presented as mean ± SD (*n* = 3 biological replicates, all averages of three technical replicates). All data were analyzed using a two-tailed *t*-test and no significant differences were found (*α* = 0.05).

To evaluate the impact of the polymerization process on cell physiology, we first assessed potential cytotoxic effects. Cell viability was measured using a fluorescent calcein/ethidium homodimer I assay, in which live cells were stained green and dead cells red. No significant difference in viability was observed between the untreated control group (91.78% ± 4.68) and the polymer-coated group (92.93% ± 5.60), as shown in Fig. 7A. The toxicity profiles of the monomer and H_2_O_2_ have been previously established in our earlier work.^21^ A representative fluorescence image (Fig. 7B) further confirmed this finding; even cells visibly coated with polymer (indicated by white arrows) remained viable immediately following polymerization.

To determine whether the polymer coating affected cellular excitability, calcium imaging was performed. Live cells were stimulated with a depolarizing concentration of KCl, and intracellular calcium dynamics were monitored. The proportion of responsive cells did not differ significantly between untreated controls (89.83% ± 5.04) and polymer-coated cells (88.16% ± 9.09), indicating that the coating did not impair the cells’ ability to respond to stimulation (Fig. 7C). Additionally, no significant difference was observed in the magnitude of calcium responses, as measured by the fluorescence ratio (*F*_max_/*F*_0_), further suggesting that cellular functionality remained intact after polymerization (Fig. 7D). These results are comparable with the results obtained in our previous work where we used an anchor-molecule to attach the polymer to the cell.^21^

To evaluate longer term effects of the polymer coating on cellular behavior, we monitored the cells for up to 96 hours following polymerization. At this time point, the polymer remained present on the cell surface, and the cells continued to migrate and divide (Fig. S11). No significant changes in migration speed were observed between polymer coated cells and control cells (Fig. S12).

## Conclusions

In summary, we present a simplified and versatile method for localized polymerization of conductive polymers on cell membranes, eliminating the need for genetic modification and its associated ethical considerations. The protocol is time-efficient and is easily transferable across different cell lines. Our findings confirm the successful formation of conductive pETE-S on the cell surface, with preserved cellular viability and excitability. Despite the limitations of heterogeneous coverage and limited electrical characterization, overall, this method provides a promising platform for the development of biointegrated electronic interfaces with minimal biological disruption.

## Materials and methods

### Materials

Sodium 4-(2-(2,5-bis(2,3-dihydrothieno[3,4-*b*][1,4]dioxin-5-yl)thiophen-3-yl)ethoxy)butane-1-sulfonate (ETE-S) was synthesized as previously described.^26^ H_2_O_2_ and HRP (type I, 107.86 U mg^−1^ solid), were purchased from Merck. All commercial chemicals were used as received without further purification.

### Cell culture

PC12 (ECACC catalog no. 88022401, RRID:CVCL 0481) cells were purchased from Ramcon (Denmark). They were cultured in Gibco's RPMI 1640 with l-glutamine (Thermo Fisher Scientific, cat. no. 21875034), supplemented with 10% Gibco's horse serum (HS, Thermo Fisher Scientific, cat. no. 16050122) and 5% Gibco's fetal bovine serum (FBS, Thermo Fisher Scientific, cat. no. 26140079). The cells were grown at 37 °C in a humidified atmosphere with 5% CO_2_ and were passaged every other day.

The F11 cell line was a gift from M. Pohl (Université Pierre et Marie Curie 6, Paris, France) and C. Svensson (Karolinska University, Stockholm, Sweden). They were cultured in Ham's F-12 Nutrient Mix GlutaMAX medium (Invitrogen, Thermo Fisher Scientific, cat.no. 31765035) supplemented with 15% Gibco's fetal bovine serum (FBS, Thermo Fisher Scientific, cat. no. 26140079), 1× sodium hypoxanthine, aminopterin, and thymidine (HAT, Invitrogen, Thermo Fisher Scientific, cat. no. 21060017), and 200 µg mL^−1^ allo-4-hydroxy-l-proline (Sigma-Aldrich). The cells were grown at 37 °C in a humidified atmosphere with 5% CO_2_ and medium was changed every other day. When 70 to 75% confluent, the cells were detached with EDTA and split in a 1 : 5 ratio.

Citrate-treated bovine whole blood was obtained from Håtunalab AB (Bro, Sweden) and separated by centrifugation at 3000 rcf for 10 min. Serum and buffy coat was removed by pipetting and the remaining RBCs were resuspended in PBS and stored at 4 °C.

SH-SY5Y cells were obtained from AddexBio (NordicBiosite, Täby, Sweden) and cultured in DMEM/F12 1 : 1 (Thermo Fisher Scientific cat. no. 11554546) supplemented with 10% FBS (Thermo Fisher Scientific, cat. no. 26140079) at 37 °C, 5% CO_2_, humidified atmosphere. Cells were detached by trypsinization (0.05% trypsin-EDTA) and split 1 : 10 once a week (at approx. 80% confluent) and received new medium every 2–3 days.

### Polymerization of pETE-S on cell membranes

All solutions for F11 cells were prepared in unsupplemented Ham's F-12 Nutrient Mix GlutaMAX medium, while solutions for PC12 cells and RBCs were prepared in unsupplemented Gibco RPMI 1640 with l-glutamine, and SH-SY5Y solutions were prepared in unsupplemented DMEM/F12 (1 : 1) medium, unless otherwise stated. F11 and SH-Sy5y cells were detached from the culture flask and collected into centrifuge tubes; PC12 cells were collected directly. All cell types were centrifuged at 200 rcf for 5 minutes, after which the supernatant was discarded. The resulting cell pellets were resuspended in 3 mL of HRP solution (107.86 U mL^−1^) and incubated at 37 °C for 30 minutes. During incubation, the tubes were placed at a 15° angle on a sunflower shaker (34 rpm) to maximise the HRP-cell interaction. Following this step, the samples were centrifuged again at 200 rcf for 5 minutes, and the supernatant was replaced with a polymerization solution containing 260 µM ETE-S and 175 µM H_2_O_2_. The cells were incubated for 45 minutes whilst shaking. After polymerization, the samples were centrifuged at 200 rcf for 5 minutes and finally resuspended in the supplemented culture medium, as described in the cell culture subsection, for subsequent experiments.

### Cell fixation for microscopy and absorbance microscopy

For PC12, coverslips were coated with 0.01% poly-l-lysine (PLL) for 10 min before the cells were added (200 000 cells per well). For F11 and SH-SY5Y, the coverslips were coated overnight with 20 mM poly-d-lysine (PDL, Sigma-Aldrich, P7280), and 3 mM laminin (Sigma-Aldrich, L2020) at 4 °C before the cells were added (25 000 cells per well). After treatment, cells were treated for 10 min with methanol-free 4% paraformaldehyde (Thermo Fisher Scientific). After incubation, the cells were rinsed three times and then mounted on microscopy slides for imaging. The cells were imaged in bright-field mode in a LSM 980 (Zeiss) fitted with a color camera. Images were post-processed to enhance the contrast to improve visibility.

### Spectroscopic analysis

Absorbance microscopy was obtained using a Nikon Eclipse L200 N optical microscope (halogen lamp 50 W). Transmitted light was collected from the camera with a guiding light (optical fiber 200 µm) connected to a spectrophotometer (OceanOptics, HR 4000, USA), and the absorption spectra (300 to 1000 nm) of selected regions were acquired. Collected spectra were averaged from five successive spectra obtained with an integration time of 100 ms. Data points under 400 nm and above 900 nm were removed, because they are at the edge of the range of the spectrometer and induced a lot of noise. Ten different cells were measured, and the resulting spectra were normalized and averaged.

### HRP quantification

Two distinct assays were used, one to visualize and the other to quantify HRP. To visualize HRP, we used a 3,3′-diaminobenzidine (DAB) staining kit (Thermo Fisher, cat. no. 8801-4965-72). For that, we incubated the cells with HRP as described previously. Next, we centrifuged the sample, resuspended them in unsupplemented cell medium and centrifuged a second time. The second wash step was found to be necessary to avoid complete consumption of DAB substrate by HRP still in the solution, which hindered imaging. The cells were then resuspended in PBS, diluted for easier cell counting and seeded into a clear 96-well plate. Next, the cells were incubated for 20 minutes with 35 µL DAB working solution/well. The stained cells were imaged in bright-field mode in a LSM 980 (Zeiss) fitted with a color camera. One photo from each well was analyzed as an RGB image in ImageJ and the red channel intensity of 15 cells per image was measured using the ellipse selection and histogram tools. Measurements from each biological replicate were normalized to an internal control without DAB staining. DAB-stained cells without HRP exposure showed no significant difference in intensity to the no-DAB controls (*P* = 0.84, one-way ANOVA). To estimate the number of cells effectively coated with HRP, one image from each biological replicate was used and 100 cells per image were manually counted as either stained or unstained.

To quantify the HRP adsorbed to the cells, we analyzed the waste solution using Neogen's 3,3′,5,5′-tetramethylbenzidine (TMB) assay (Thermo Fisher, cat. no. 50-134-9564). After HRP incubation and centrifugation, the supernatant was collected. This was diluted 10 000×, and mixed in equal parts with the TMB solution (which already contained H_2_O_2_). The reaction was stopped by adding 2M H_2_SO_4_ after 60 seconds. Absorbance measurements at 450 nm were performed in a Synergy H1 plate reader (BioTek), operating at room temperature.

### Scanning electron microscopy

Gold-coated glass coverslips were prepared by thermal evaporation with 5 nm of Cr and 45 nm of Au. Then, the gold-coated coverslips were treated with ultraviolet (UV)–ozone (PSD-UV8, Novascan) for 30 min and immediately soaked in DI water. After 10 min of subsequent UV-C treatment for sterilization purposes, the coverslips were coated as previously described for microscopy samples. Polymer-treated cells and untreated control cells were seeded overnight on the coverslips (PC12: 200 000 cells per well; F11: 25 000 cells per well). Next, cells were treated for 2 h with 2.5% glutaraldehyde solution (Thermo Fisher Scientific) and 2% methanol-free paraformaldehyde (Thermo Fisher Scientific). Next, the cells were rinsed three times for 10 min with 0.1 M sodium cacodylate buffer (Thermo Fisher Scientific). This was followed by a secondary lipid fixation step by incubating the samples in 0.5% osmium tetroxide (Thermo Fisher) in sodium cacodylate buffer for 2 h and then rinsed three times for 5 minutes with DI water. Next, the cells were dehydrated using the following series: 50% ethanol for 5 min; 70% ethanol for 5 min; 95% ethanol for 10 min; and twice 100% ethanol for 10 min. Once dehydrated, the ethanol solution was replaced with 100% hexamethyldisilazane (HMDS, Thermo Fisher Scientific) for 3 min. Finally, the HMDS was removed, and samples were left overnight to let the remnants evaporate.

After this, the cells were sputter-coated (Leica EM ACE600) with a 3 nm thick layer of Au to minimize charging during imaging. The morphology and chemical composition of the cells and the polymer coatings were studied using an SEM (JEOL 7800F Prime) at high-vacuum conditions. Information about the surface morphology was obtained using the secondary electron signal for imaging at an acceleration voltage of 1 kV. Information about the chemical composition was obtained using energy dispersive X-ray spectroscopy (EDX) (Oxford X-Max 80 mm2 detector) at an acceleration voltage of 5 kV and 10 kV.

Quantitative characterization of the cellular and polymer coating morphologies was performed based on SEM images of 100 F11 cells using ImageJ (National Institutes of Health; available at https://imagej.net/). The cell area was determined by manually tracing the contour of the main cell body and large outgrowths, disregarding the dendrites. The polymer coating area was determined by manually tracing the contour of all individual polymer patches contacting each cell. It should be noted that the measured areas are projected areas, which underestimate the true surface area of a cell with pronounced surface topography.

### Focused ion beam (FIB)-scanning electron microscopy

F11 control cells without polymer and polymer-treated cells were cross-sectioned using FEI Versa3D and Tescan GAIA3 FIB-SEM instruments to reveal the internal microstructure and determine the thickness of the polymer coatings. A strip of platinum was deposited in the FIB-SEM instruments locally before milling in order to protect the surface structure and reduce curtaining effects, as has been shown in previous works.^27–29^ The cross-sections were imaged with SEM (JEOL 7800F Prime) using secondary electrons and backscattered electrons at an acceleration voltage of 5 kV.

### Conductive AFM

Samples were prepared similarly to SEM until the first fixation step (2% PFA and 2.5% glutaraldehyde). After that, samples were rinsed three times with DI and dried using nitrogen gas right before measurements.

C-AFM was performed in a Dimension Icon XR microscope (Bruker) operating in PF-TUNA mode. Height images and current maps were collected in PeakForce Tapping mode with Pt/Ir-coated silicon probes having a spring constant of 3 N m^−1^, a resonance frequency of 75 kHz, and a nominal tip radius of 25 nm. For current maps and local *I*–*V* curve acquisitions, a DC bias was applied to the conductive substrate while keeping the probe at ground.

### Toxicity tests

To examine the cytotoxic effects of the polymerization process on F11 cells, a fluorescent live/dead viability kit for mammalian cells was used (Thermo Fisher Scientific, catalog no. L3224). A 96-well plate was coated overnight with 20 mM PDL (Sigma-Aldrich, P7280) and 3 mM laminin (Sigma-Aldrich, L2020) at 4 °C. Next, the cells were subjected to the polymerization process, and 10 000 cells were seeded per well and left overnight for attachment. The toxicity was assessed using the kit according to the manufacturer's instructions. The cells were imaged using a ZOE fluorescent cell imager (Bio-Rad Laboratories). We obtained three biological replicates per test and three technical replicates.

### Calcium imaging

Cells were prepared as described for the toxicity tests, except in a LUMOX well plate. The calcium-sensitive fluorescence indicator Fluo-4AM (Thermo Fisher Scientific, 5 µM) was used and the cells were loaded for 30 to 40 min (RT). Cells were washed with modified HEPES buffer (145 mM NaCl, 3 mM KCl, 2 mM CaCl_2_, 2 mM MgCl_2_, 10 mM glucose, and 10 mM HEPES, pH 7.4, with NaOH). A Zeiss LSM980 inverted confocal microscope was used with a 20× objective to carry out calcium imaging experiments. First, a baseline was established for approximately 10 s, after which 30 mM KCl was added. The fluorescent response was monitored for about 1 min with approximately one picture every second. ImageJ software was used to analyze the images. All visible cells in each image were selected for measurement of mean fluorescence intensity (*F*) in the region of interest (the cell bodies). Data are presented as *F*_max_/*F*_0_ with *F*_0_ being the average cell intensity from the first 10 images in each series. Cells were considered positive responders if they showed an increase of at least 25% of *F* compared to *F*_0_.

### Characterization of migration

F11 cells were polymerized as described above and seeded in a PDL/laminin-coated chamber slide overnight. After that, the cells were placed in the LSM 980 (Zeiss) fitted with a color camera and an incubator (37 °C, 95% humidity and 5% CO_2_). Cells were imaged over 24 h with a picture every 15 min. The data obtained from three biological replicates were analyzed using the manual tracking plugin in ImageJ.

### Statistics

Statistical analyses were performed using GraphPad Prism 10.0.2. If two groups were compared, a two-tailed *t* test was used and *p*-values less than 0.05 were considered significant.

## Conflicts of interest

There are no conflicts to declare.

## Supplementary Material

MH-013-D5MH02264A-s001

## Data Availability

The data that support the findings of this study are available in the paper and/or the supplementary information (SI). Supplementary information is available. See DOI: https://doi.org/10.1039/d5mh02264a. Additional data are available from the corresponding author upon reasonable request.
