# Supplementary material for: Suspension polymerization of bioelectronic interfaces on living cells
Source: Mater Horiz. 2026 Mar 26;13(12):5888–97. doi: 10.1039/d5mh02264a (PMC13036600; doi:10.1039/d5mh02264a)
Supplement: MH-013-D5MH02264A-s001 [file MH-013-D5MH02264A-s001.pdf]

Supplementary Information (SI) for Materials Horizons.
This journal is © The Royal Society of Chemistry 2026

SUPPLEMENTARY INFORMATION

Suspension polymerization of bioelectronic interfaces on living cells

Hanne Biesmans, †,[a] Charlotte Theunis, †,[a] Rebecka Rilemark, [b] Caroline Lindholm,[a] Marle E.J. Vleugels,[a] Tobias Abrahamsson,[a] Xenofon Strakosas,[a] Jennifer Y. Gerasimov,[a] Daniel T. Simon,[a,*] Magnus Berggren,[a, c] Eva Olsson,[b] and Chiara Musumeci.[a,*]

[a] Laboratory of Organic Electronics, Department of Science and Technology, Linköping University, 601 74 Norrköping, Sweden

[b] Department of Physics, Chalmers University of Technology, 41296 Göteborg, Sweden

[c] Wallenberg Initiative Materials Science for Sustainability, Department of Science and Technology, Linköping University, 601 74, Norrköping, Sweden

† These authors contributed equally

*Corresponding authors: daniel.simon@liu.se, chiara.musumeci@liu.se

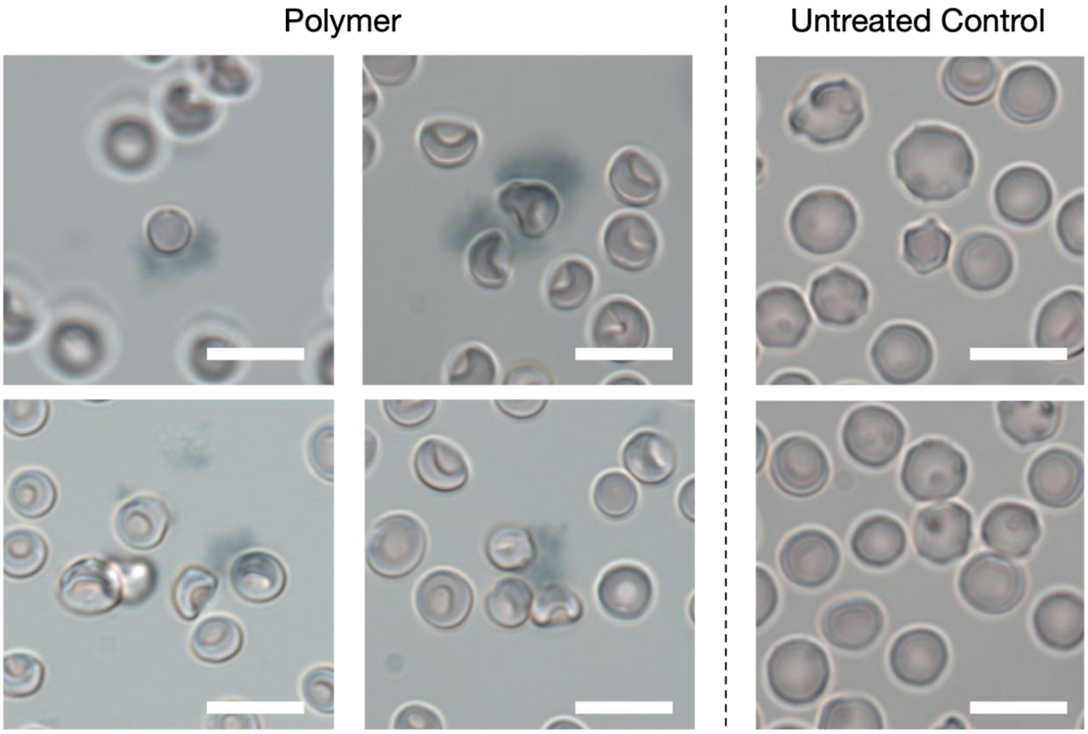

Figure S1. Suspension polymerization on Red Blood Cells. Scale bars are 10 µm.

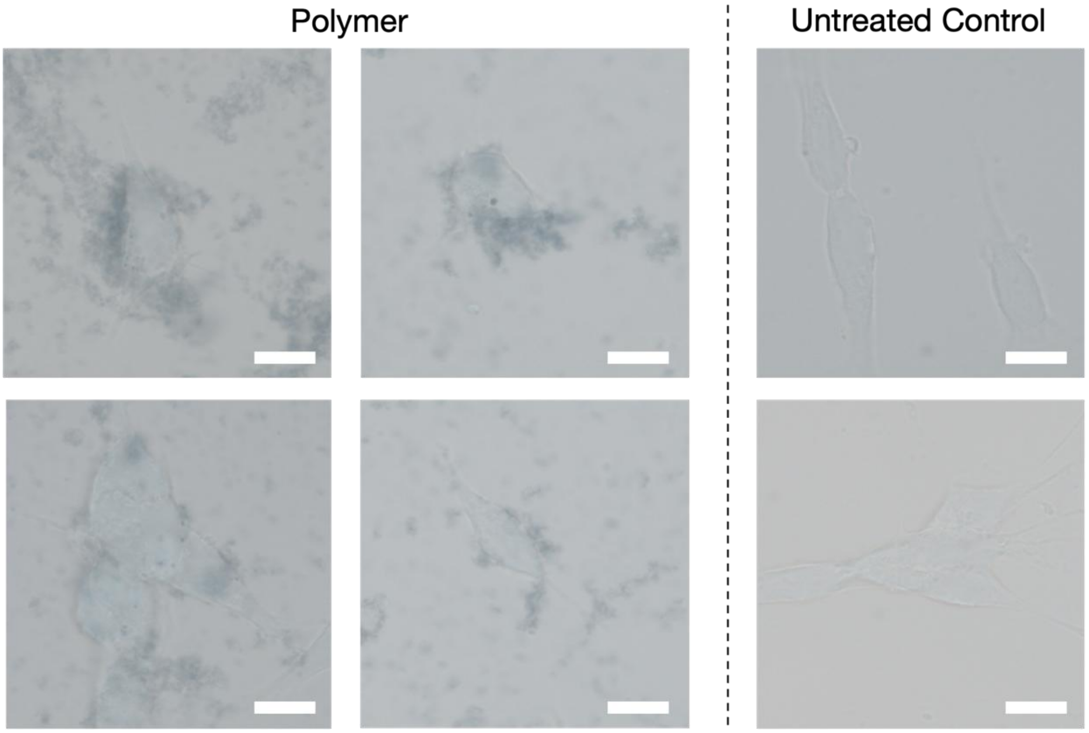

Figure S2. Suspension polymerization on SH-Sy5y cells. Scale bars are 10 µm.

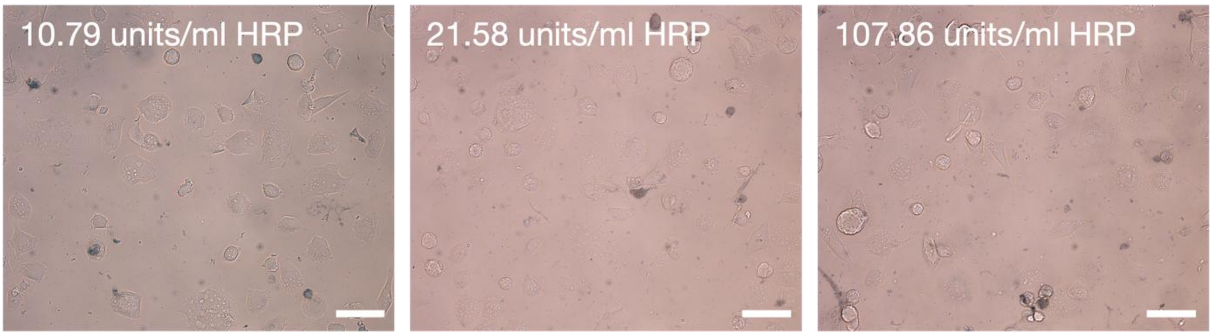

Figure S3. One-step polymerization of ETE-S with different concentrations of HRP. F11 cells in suspension were mixed with 260 µM ETE-S, 175 µM H_2O_2 and different concentrations of HRP while shaking. Only the smallest cells were coated with polymer. Scale bars are 50 µm.

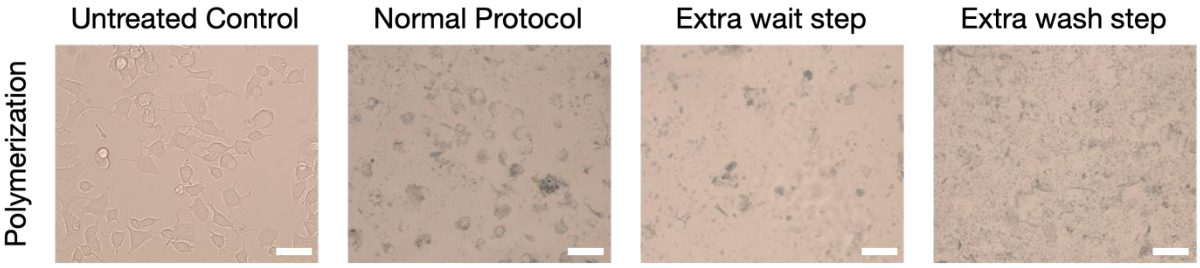

Figure S4. Diminished cell targeting due to HRP release. Extended waiting time or an extra wash step after HRP incubation results in the formation of less cell-templated polymer and more non-specific polymer. Scale bars are 50 µm.

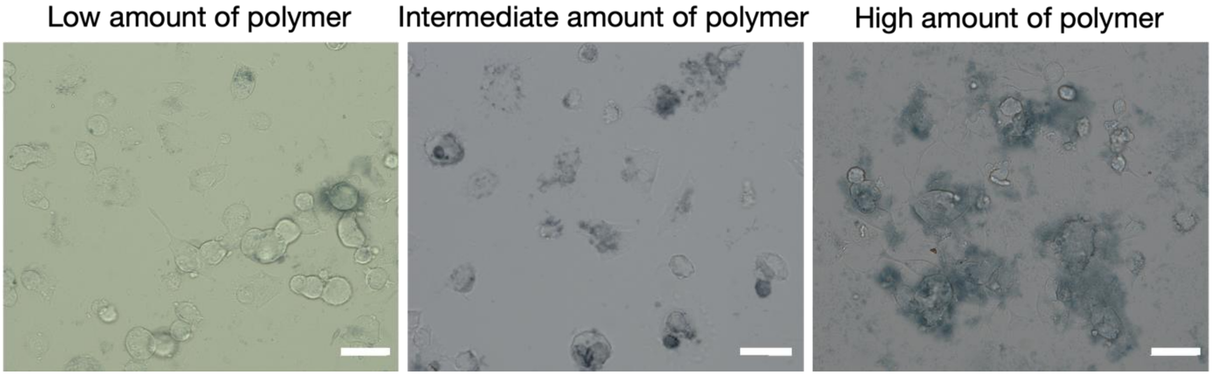

Figure S5. Variability in polymerization efficiency. Application of an identical polymerization protocol yields a broad range of polymerization outcomes, from minimal to extensive polymer deposition. Scale bars represent 50 µm.

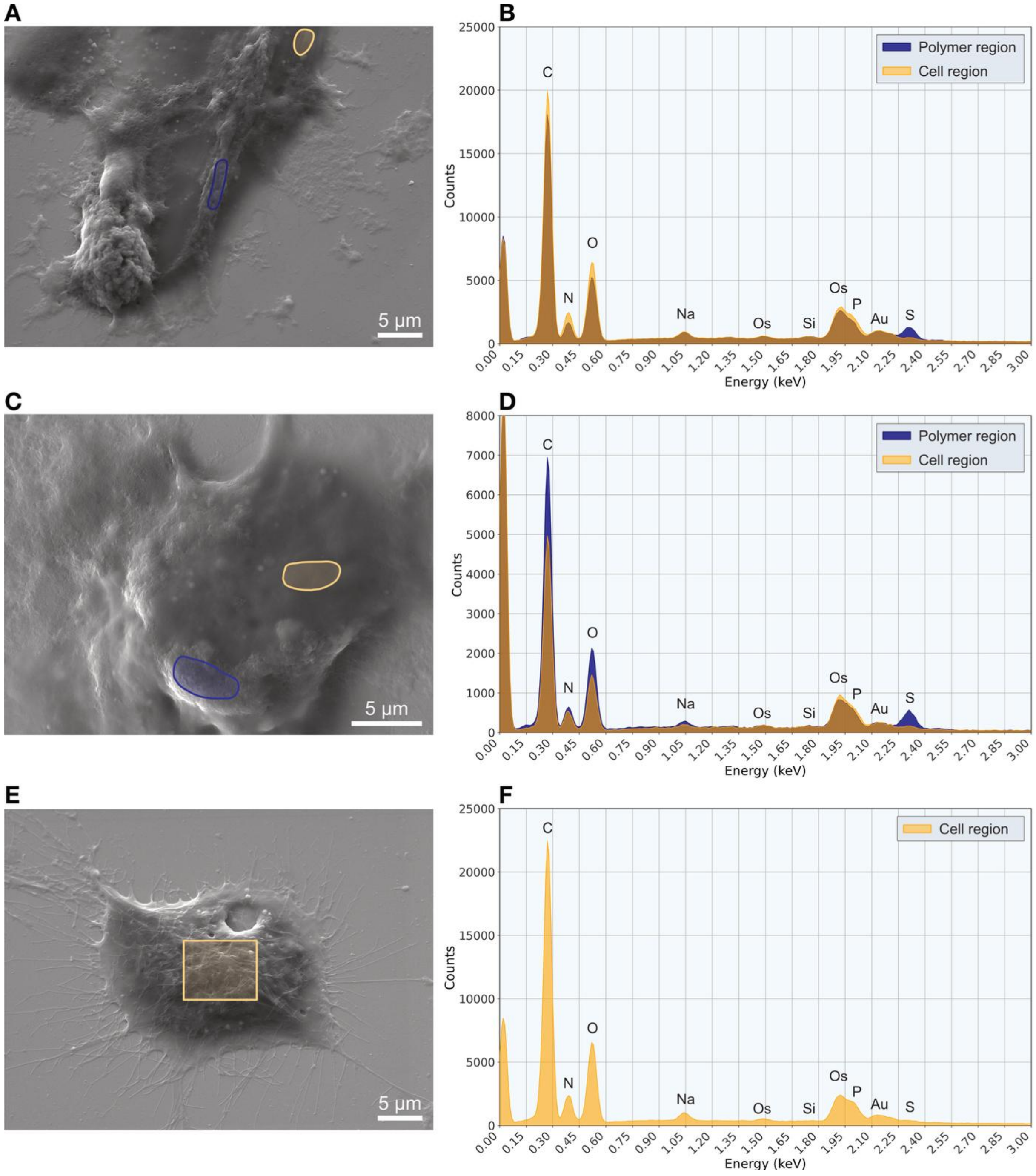

Figure S6. Analysis of the chemical composition of F11 cells after suspension polymerization (A-D) and F11 cells without polymer treatment (E,F). (A, C, E) SEM secondary electron images taken at 10 kV of F11 cells. (B, D, F) EDX spectra. Blue spectra are acquired from regions showing surface morphology attributed to the polymer coating (blue regions). Yellow spectra are acquired from regions of cell surface without polymer coating (yellow regions). The blue regions contain a higher amount of sulfur compared to the yellow regions, which is attributed to the sulfonate group and the thiophene backbone of the polymer present in these regions.

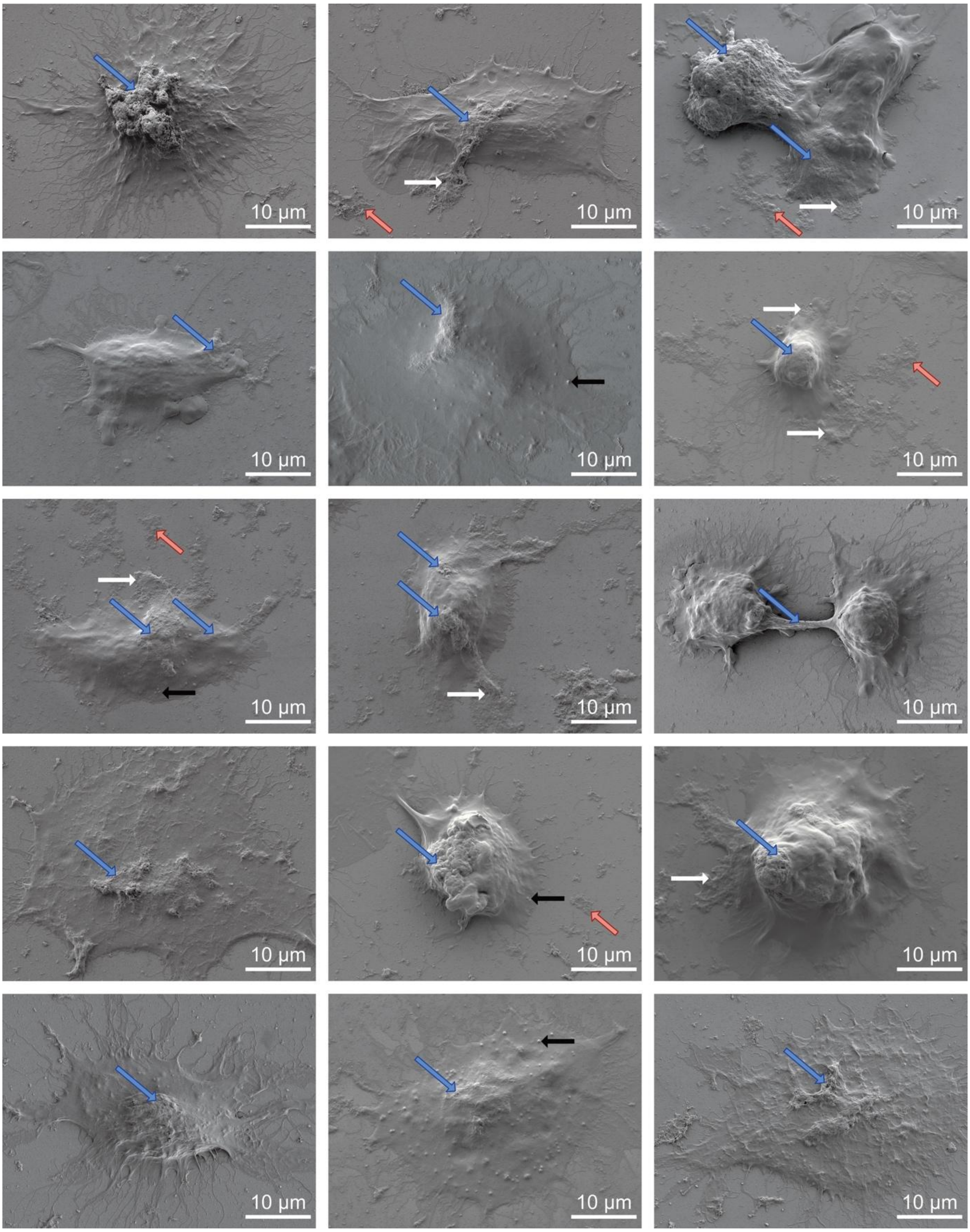

Figure S7. SEM secondary electron images of F11 cells polymerized in suspension. The images show examples of cells included in the quantification of polymer coverage in Figure 4. Blue arrows point to examples of PETE-S coatings on the cells. White arrows point to examples of patches that are in contact with the cells but also extend beyond the main cell body. Red arrows point to pETE-S patches on the surrounding substrate, which are not included in the quantification. Black arrows point to spherical vesicles that are 200-700 nm in diameter.

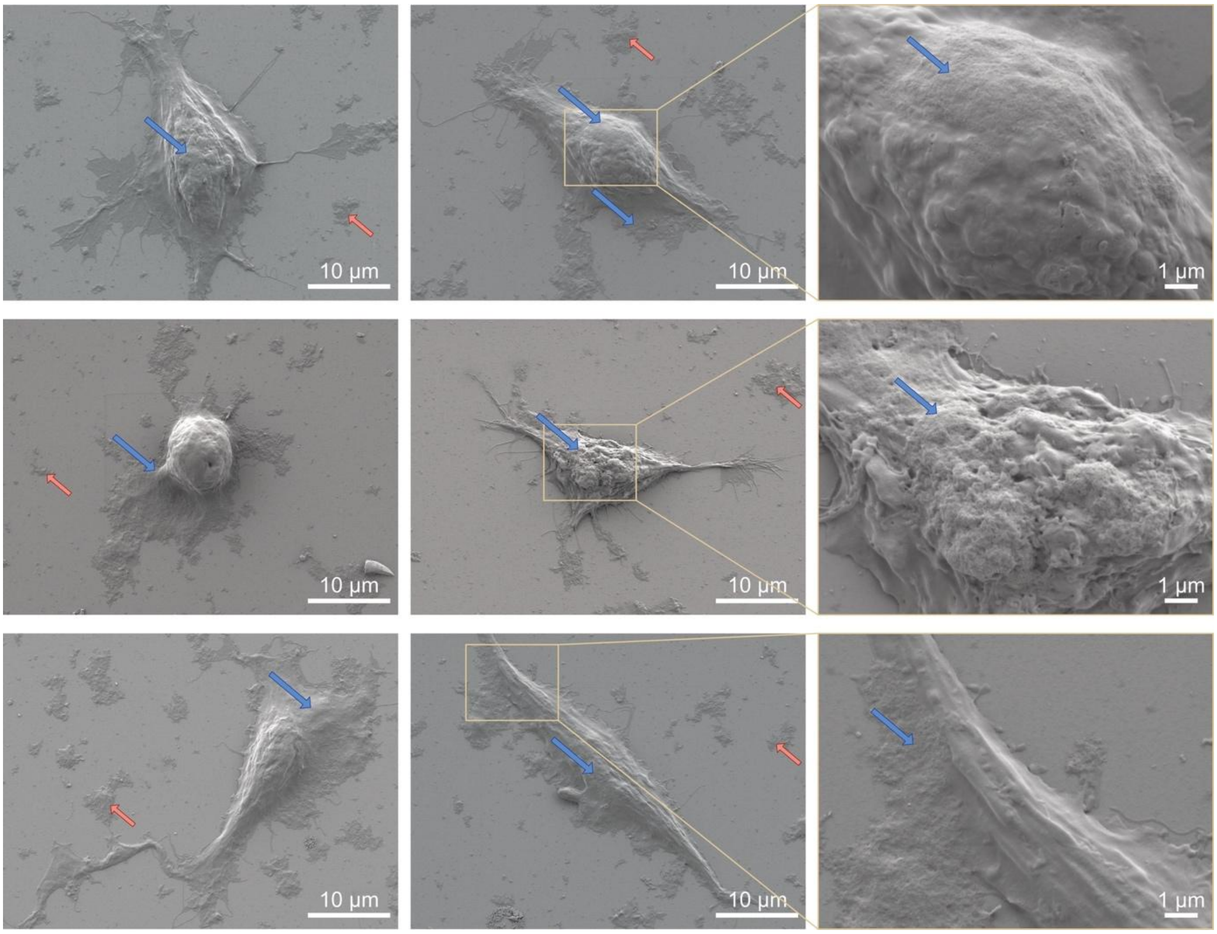

Figure S8. SEM secondary electron images of PC12 cells after suspension polymerization. Blue arrows point to examples of pETE-S coatings located entirely or partially on the cells. Red arrows point to pETE-S patches on the surrounding substrate. The right column shows higher magnification images of the corresponding regions marked in the middle column.

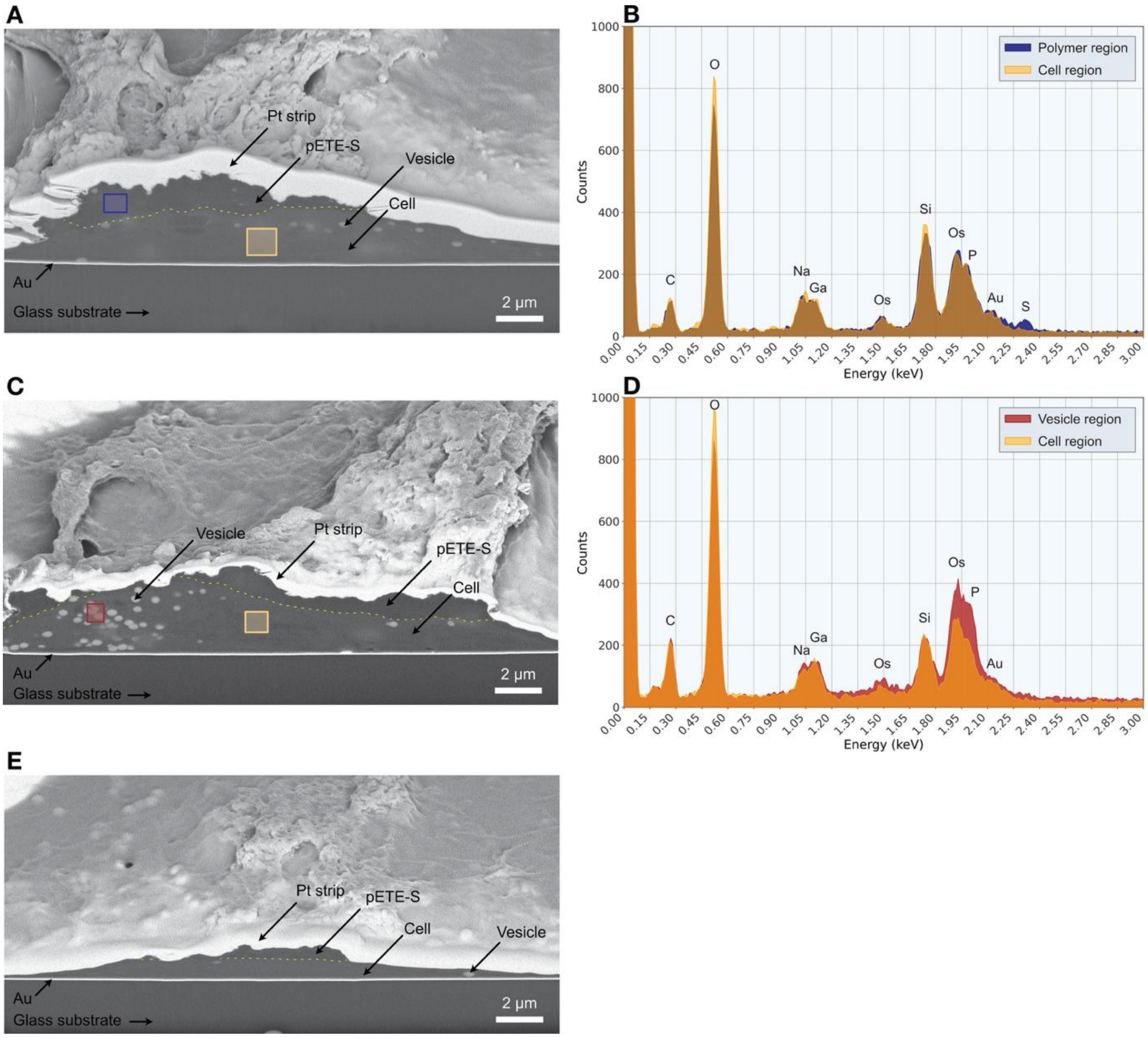

Figure S9. Identification of polymer part in cross sections. (A, C, E) SEM backscattered electron images taken at 5 kV of FIB cross sections of F11 cells with pETE-S coatings. The cross sections are viewed at an angle of 45° relative to the cross section surface. The polymer coating is identified as the region with dark contrast directly below the protective platinum strip. The dashed line marks the interface between the polymer and the cell. Vesicles are found within the cell region as bright, round particles with diameters in the range of 200-700 nm. (B) EDX spectrum from the polymer region (blue) in (A) overlaid with an EDX spectrum from the cell region (yellow). The polymer region contains a higher amount of sulfur than the cell region. (D) EDX spectrum from a vesicle-rich region (red) in (C) overlaid with an EDX spectrum from the cell region (yellow). The osmium and phosphorus contents are higher in the vesicles than in the surrounding cell.

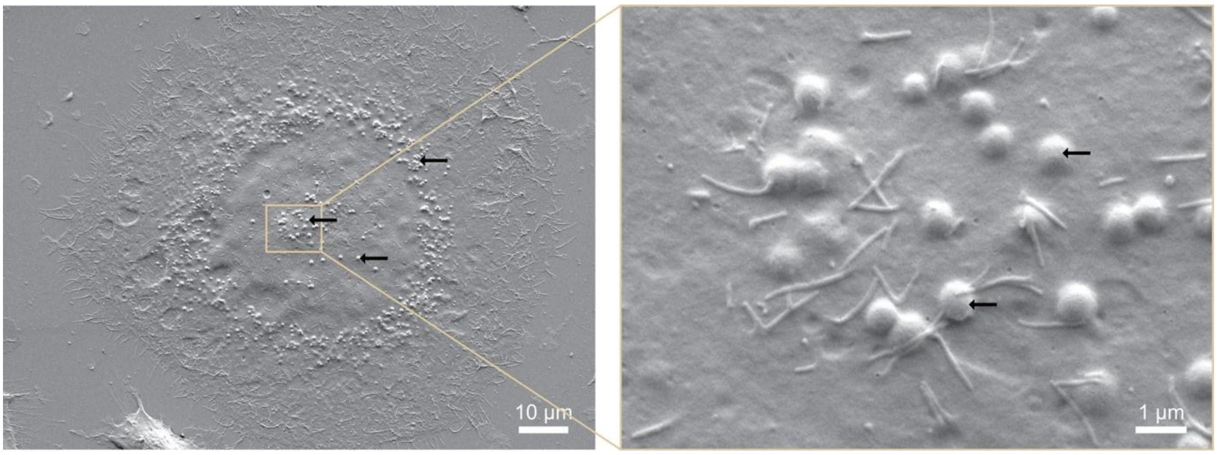

Figure S10. SEM secondary electron image of the surface morphology of an untreated F11 control cell with spherical vesicles (black arrows). The vesicles have diameters in the range of 200-700 nm.

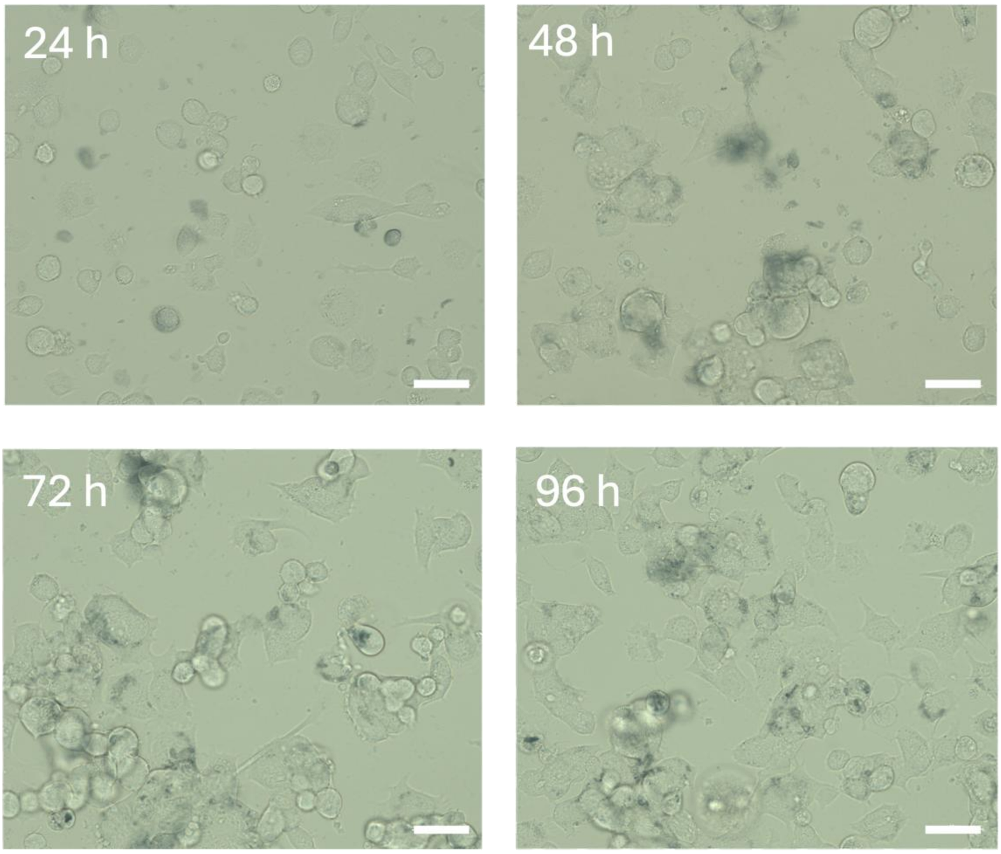

Figure S11. Polymer stability over time. 1 position with F11 cells imaged 24 h, 48 h, 72 h, and 96 h after polymerization. Scale bars represent 50 µm.

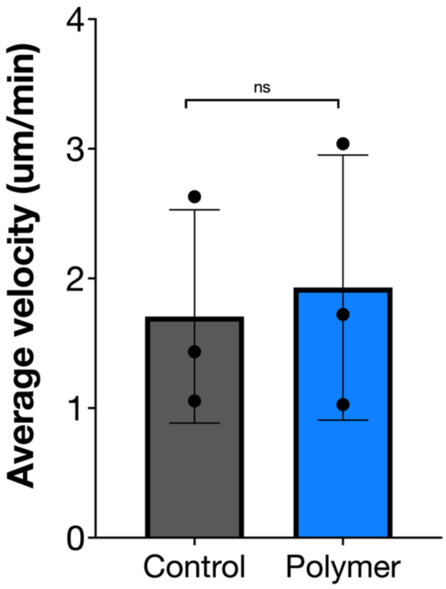

Figure S12. Migration speed of polymer coated cells versus untreated control cells. Polymer coated and untreated control cells were followed overnight to determine their average migration velocity. Data is presented as mean ± SD (n = 3 biological replicates, all averages of three technical replicates). Statistical analysis was performed using a two-tailed t-test with α = 0.05 (*, p=0.0310).

Table S1. Comparison between different cell-templating polymerization methods

	Suspension Polymerization	Anchor-based Polymerization (21)	Genetic Engineering	
			Non-membrane targeted enzymes (16)	*Membrane-targeted enzymes (17)*
**Basic principle**	HRP interacts with the membrane without genetic modifications to polymerize the polymer directly onto the cell.	Anchor molecule ETE-PEGO was inserted in the membrane to attach polymer to the cell	Genetically altered cells produce the enzyme Apex-2 to trigger juxtamembranous polymerization of PANI	Genetically altered cells produce the enzyme HRP bound to the membrane to trigger polymerization of PANI on the membrane
**Cell types**	PC12; F11; RBCs; SH-Sy5y	PC12; F11	Primary postnatal hippocampal rat neurons; human embryonic kidney (HEK) 293T	Primary postnatal hippocampal rat neurons
**Specificity**	Non-specific	Non-specific	Targets specific cells	Targets specific cells
**Coating morphology**	○ Continuous polymer patches<br>○ 24% coverage<br>○ Size: majority 0.01 μm^2 – 10 μm^2; outliers to 850 μm^2	○ Continuous polymer patches<br>○ Coverage not quantified<br>○ Size: not quantified	○ Dispersed polymer aggregates<br>○ Coverage not quantified<br>○ Size: not quantified	○ Dispersed polymer aggregates<br>○ Coverage not quantified<br>○ Size: 120 ± 13 nm
**Cytotoxicity**	Viability: 92.93 ± 5.6% (F11)	Viability: 94.77 ± 0.97% (F11)	Not quantified	Not quantified
**Electrical characterization**	> 5 nA (200 mV) measured with cAFM on an individual cell (untreated)	~ 4 nA (800 mV) measured with cAFM on an individual cell (untreated)	Up to 20 nA (100 mV) with IV measurements on a subconfluent cell layer (doped with HCl vapor)	Not reported
**Operational complexity**	Low	Low	Genetic modification = high	Genetic modification = high
**Time from pristine to polymer-functionalized cell**	~ 2 hours	~ 6 hours	10-13 days (6-7 days for transfection; 4 -6 extra days before use)	10-13 days (6-7 days for transfection; 4 -6 extra days before use)
